# Slow Thinking for Fast Results: Reclaiming Analytical Reasoning in Antimicrobial Susceptibility Test Interpretation

**DOI:** 10.1093/ofid/ofag214

**Published:** 2026-04-24

**Authors:** Carlos R V Kiffer, Paulo J M Bispo

**Affiliations:** Infectious Diseases Discipline, Grupo de Análises em Infecções e Antimicrobianos (GAIA), Laboratório Especial de Microbiologia Clínica—LEMC), Antimicrobial Resistance Institute of São Paulo (ARIES), Escola Paulista de Medicina (EPM), Universidade Federal de São Paulo—UNIFESP, São Paulo, Brazil; Department of Ophthalmology, Massachusetts Eye and Ear, Infectious Disease Institute, Harvard Medical School, Boston, Massachusetts, USA

**Keywords:** antibiograms, antimicrobial drug resistance, cognition, cognitive training, psychological processes

## Abstract

Antimicrobial susceptibility testing (AST) is the basis of directed antimicrobial therapy and stewardship. However, its interpretation frequently relies on rapid and intuitive judgment, rather than on deliberate analytical reasoning. Using Kahneman's dual-process theory of cognition, we argue that AST interpretation is particularly vulnerable to system 1 heuristic bias, especially in real clinical environments. Using illustrative examples from contemporary resistance scenarios, such as extended-spectrum β-lactamase–producing Enterobacterales and multidrug-resistant *Pseudomonas aeruginosa*, we discuss the evolving resistance patterns over years, and it has increased interpretive complexity beyond what categorical AST outputs alone convey. We propose that interpretative errors may often arise from predictable cognitive responses, rather than from insufficient knowledge, which may be reinforced by current laboratory reporting formats and clinical workflows. Finally, we discuss educational, structural, and reporting strategies to foster system 2 engagement and mechanism-aware interpretation, with implications for antimicrobial stewardship and the training of medical students and early-career clinicians.

## Teaser

This viewpoint explores how evolving antimicrobial resistance complicates susceptibility testing interpretation, increases cognitive complexity and heuristic bias in light of Kahneman's dual-process theory, arguing that educational and structural interventions are necessary to strengthen analytical reasoning in antimicrobial therapy decision processes.

Few clinical tasks appear as routine as reading an antimicrobial susceptibility test (AST) report. Antimicrobial susceptibility test reports listing categorical results such as susceptible (S), intermediate (I), or resistant (R) typically invite a rapid recognition pattern and swift therapeutic decisions. This apparent simplicity is misleading, since it involves substantial interpretive complexity.

Antimicrobial susceptibility test results represent probabilities of therapeutic outcomes based on antimicrobial resistances (AMRs), pharmacokinetics/pharmacodynamics (PK/PD), and infection characteristics. As such, it requires deliberate analytical thinking. However, simplified results presented solely as S/I/R may trigger rapid and intuitive thinking. This rapid thinking increases the vulnerability of cognitive bias, potentially leading to interpretative errors that may directly influence outcomes, such as the overuse of antimicrobials or the use of inappropriate therapies and doses that can result in increased mortality [1–3].

Clinical reasoning has been described as the dynamic interaction between fast pattern recognition (system 1 or S1) and slower deliberate analytical reasoning (system 2 or S2), based on Kahneman's model [4]. System 1 enables rapid decision-making but is vulnerable to anchoring and premature closure biases. Anchoring occurs when clinicians rely excessively on initial information, such as selecting antimicrobials based solely on simple categorical AST results. Premature closure happens when therapy is chosen without fully evaluating the clinical, microbiological, and PK/PD dimensions. Ideally, S2 should supervise S1 in interpreting AST results to ensure these critical aspects are integrated. This cognitive model provides an interesting framework for understanding vulnerabilities in AST interpretation, a task particularly susceptible to interactions between these systems.

This perspective examines the premise that AST interpretation is cognitively vulnerable, illustrates these vulnerabilities using common AMR scenarios, and discusses practical strategies to support more deliberate clinical thinking in AST interpretation.

In routine practice, AST interpretation may be impacted by results presented as simple categorical outputs, without minimum inhibitory concentrations (MICs), PK/PD guidance, or indication of likely resistance mechanisms. Heterogeneity in AST reports across practices may amplify this effect [5]. This variability constitutes a structural determinant of cognition, since interfaces that compress multidimensional microbiological information into S/I/R labels favor fast interpretation and reduce cognitive cues for slow analytical cross-checking. Thus, simplified AST results may activate S1 thinking based on the shortcut “S = good, R = bad,” particularly attractive under workload pressure, limited microbiological expertise, or high patient volumes.

Cognitive vulnerabilities are accentuated by the complexity of antimicrobial PK/PD contexts associated with AST interpretations. In practice, the clinical meaning of susceptible depends on whether a given regimen can achieve therapeutic levels in the infected site [5, 6]. This is important in systemic treatments when optimized dosing can enhance drug penetration and also relevant for localized infections, such as those of the skin, eye, ear, or bladder, where novel or optimized drug-delivery systems can result in local concentrations that are orders of magnitude higher than those attainable with systemic dosing [7–9]. While these innovations expand therapeutic options, they also demand deliberate reasoning (S2) about route of administration, formulation, and pharmacokinetics [10–12]. A purely heuristic (S1) reading of categorical outputs, detached from the exposure assumptions underlying that classification, risks both underuse of potentially effective high-exposure local strategies or high-dose systemic therapies and overconfidence in systemic regimens that cannot reproduce PK/PD targets at the infected site.

The risk of heuristic responses in AST interpretation can affect both inexperienced and seasoned clinicians that are driven to rapid recognition of AST patterns previously treated with some degree of success, particularly in environments with scarcity of experts, delayed confirmatory testing, and high therapeutic urgency. However, in the current global scenario of rapidly changing AMR epidemiology, such reliance on intuition becomes highly problematic. To illustrate this, we have selected didactic archetypes of AMR that may trigger heuristic thinking and result in recurrent cognitive failure modes.

The first archetype involves extended spectrum β-lactamases (ESBL)-producing Enterobacterales in urinary tract infections, a major driver of antimicrobial use [12]. Antimicrobial susceptibility testing reports of ESBL-producing isolates may display discordant cephalosporin susceptibility patterns, with susceptibility to one and resistance to another third-generation cephalosporin. Clinicians may anchor on the first “S” result equating it with clinical efficacy and overlook the implications of ESBL production, particularly when under pressure or in places where this phenotype is unexpected, a classic S1 shortcut. System 2 engagement would flag these discordant patterns as a conflict signal, prompting interrogation of ESBL epidemiology, breakpoint assumptions, and achievable drug exposures, recognizing that such AST patterns require S2-type interpretation of results for appropriate drug selection [2, 6, 13].

The second archetype involves multidrug-resistant *Pseudomonas aeruginosa*, particularly in settings with high prevalence of AMR. *Pseudomonas aeruginosa* is capable of accumulating AMR via multiple mechanisms that can generate overlapping phenotypic AST patterns [14–16]. For example, an isolate with OprD porin loss may be resistant to imipenem but susceptible to meropenem and could be treated with optimized meropenem dosing in the absence of carbapenemase production. In another scenario, a metallo-β-lactamase (MBL) producer may be resistant to aztreonam due to coexpression of AmpC or ESBLs, a commonly found profile [16]. Yet, aztreonam remains active against MBL, and its use in combination with avibactam or other antimicrobials may still be appropriate [17]. A simple S1-type interpretation of these results could lead to inappropriate therapy selection.

Antimicrobial susceptibility testing interpretation is a cognitive task shaped by microbiology and epidemiological context and individual expertise. Simple categorical outputs, limited contextual cues, fragmented laboratory-clinician communication, time pressure, and evolving AMR mechanisms with ambiguous phenotypes favor S1 fast thinking and can limit activation of S2 oversight.

Clinical & Laboratory Standards Institute (CLSI) and the European Committee on Antimicrobial Susceptibility Testing (EUCAST) frequently revise breakpoints to better reflect PK/PD principles, tissue penetration, and dosing strategies [18–20]. Their guidelines increasingly distinguish systemic from urinary breakpoints, specify standard versus high-dose regimens, and provide site- and route-specific interpretations. Overreliance on S1 shortcuts that treat “S” results as equivalent across tissues, routes, and doses is increasingly misaligned with this PK-/PD-informed granularity, underscoring the need for intentional S2 engagement when interpreting AST.

**Figure 1.**
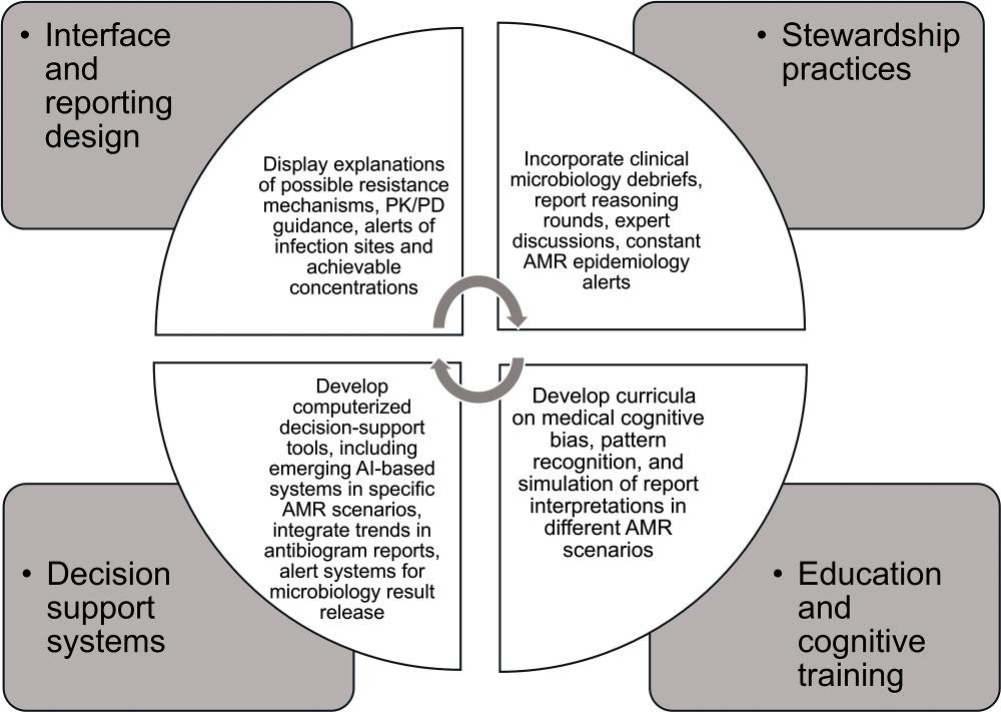
Approaches to enhance S2 reasoning in AST interpretation and clinical training.

Clinical reasoning is inherently probabilistic and time pressured. Expert clinicians do not, and should not, abandon intuitive reasoning; rather, they should develop the capacity to recognize when intuition should be supervised. The challenge lies in creating environments that reliably trigger this cognitive “shift” for AST interpretation. Engineering S2 engagement is possible, and Figure 1 outlines strategies to promote this engagement, including optimized report design, interpretive comments, stewardship actions, and advanced decision-support tools. These interventions are not intended to slow care indiscriminately, but to ensure activation of analytical reasoning when interpretive risk is high.

The present perspective does not aim to exhaust the subject or to suggest a completely new framework; rather, its purpose is to integrate cognitive theory with microbiological practice and to generate testable hypotheses with intentional interventions to engage S2 reasoning, specifically for antimicrobial stewardship and clinical training. Future research could examine these concepts with studies on time-to-decision analyses, interface-design trials, or prescribing behavior versus AST report formats. Antimicrobial susceptibility testing is a microbiological measurement and a cognitive process. The simplicity of S/I/R conceals a multidimensional decision that increasingly demands S2 engagement in an era of widespread AMR. By embedding reflection cues into laboratory interfaces, stewardship practices, and education, AST interpretation can evolve from a potentially heuristic task into a deliberately analytical process. Slow thinking, paradoxically, may be the fastest way to ensure that the right antibiotic reaches the right patient and at the right dose.
